# Changes in the Cutaneous Silent Period Following Pallidal Deep Brain Stimulation in Patients with Cervical Dystonia

**DOI:** 10.5334/tohm.1241

**Published:** 2026-10-28

**Authors:** Eiichirou Urasaki, Yasushi Miyagi

**Affiliations:** 1 Department of Neurosurgery, Fukuoka Mirai Hospital, Medical Co. LTA (Living Together Association), Fukuoka, Japan

**Keywords:** cervical dystonia, deep brain stimulation, globus pallidus internus, cutaneous silent period, motor inhibition, neurophysiology

## Abstract

**Background:**

Dystonia is associated with impaired sensorimotor inhibition across distributed neural circuits; however, whether spinal inhibitory function changes following globus pallidus internus deep brain stimulation (GPi-DBS) remains unclear. This study aimed to examine perioperative changes in spinal inhibitory function in patients with cervical dystonia undergoing GPi-DBS.

**Methods:**

We investigated cutaneous silent period (CuSP) responses in 18 patients with cervical dystonia before and after bilateral GPi-DBS and compared them with those of 18 age- and sex-matched healthy controls.

**Results:**

At the early postoperative assessment during active GPi-DBS, CuSP end latency and duration were significantly increased compared with preoperative values, whereas onset latency remained unchanged. Neither preoperative nor postoperative end latency or duration differed significantly from healthy control values. Clinical severity improved after surgery; however, changes in CuSP parameters were not significantly correlated with improvements in Toronto Western Spasmodic Torticollis Rating Scale scores.

**Discussion:**

The significant early postoperative changes in CuSP end latency and duration suggest that the CuSP in patients with cervical dystonia is not a fixed phenomenon but may change dynamically during the early postoperative period while GPi-DBS is active, although neither preoperative nor postoperative values differed significantly from those of healthy controls.

**Highlights:**

CuSP duration and end latency increased at the early postoperative assessment during active GPi-DBS, suggesting that spinal inhibitory processing may change in association with perioperative modulation of the broader motor network.

## Introduction

Dystonia is a movement disorder characterized by sustained or intermittent muscle contractions that produce abnormal movements, posture, or both. Although historically attributed primarily to basal ganglia dysfunction, contemporary evidence supports a broader conceptualization in which dystonia arises from abnormalities in distributed sensorimotor networks involving the basal ganglia, thalamus, cerebral cortex, cerebellum, and brainstem [1,2]. Within this network framework, impaired inhibitory control across multiple levels of the sensorimotor system is considered a core neurophysiological feature of the disorder [1,3]. Neurophysiological studies have demonstrated abnormalities in several cortical inhibitory mechanisms in dystonia, including intracortical and surround inhibition [1,3,4,5]. Under normal conditions, surround inhibition facilitates selective motor output by suppressing activation of adjacent or competing muscles during movement initiation, thereby refining movement execution. Impairment of this mechanism has been well documented in focal dystonia and is thought to contribute to the loss of motor selectivity that underlies abnormal muscle overflow and co-contraction. This deficit is partly related to abnormal modulation of intracortical inhibitory circuits during movement initiation [6,7].

Beyond supraspinal dysfunction, altered spinal inhibitory processing may also contribute to dystonic pathophysiology. Deficient reciprocal inhibition has long been implicated in the simultaneous activation of agonist and antagonist muscles [1]. More recently, the cutaneous silent period (CuSP) has attracted attention as a clinically accessible electrophysiological probe of spinal segmental inhibition [8,9,10]. The CuSP is a nociceptive inhibitory reflex in which activation of cutaneous Aδ afferents transiently suppresses ongoing voluntary electromyographic activity via inhibitory interneuronal circuits within the spinal cord [11,12,13]. As it reflects spinal inhibitory processing yet remains susceptible to descending modulation, the CuSP may offer a useful window into interactions between supraspinal and spinal motor networks.

However, prior studies examining CuSP abnormalities in patients with dystonia have produced inconsistent results. Some investigators reported prolonged CuSP end latency in patients with focal hand dystonia and related disorders, suggesting altered inhibitory timing [8,9]. Conversely, others found no significant CuSP latency abnormalities in patients with cervical or generalized dystonia and observed no immediate changes after short-term activation or deactivation of globus pallidus internus deep brain stimulation (GPi-DBS) [10]. Gündüz et al. reported that the end latency of the mixed nerve silent period in patients with cervical dystonia did not differ significantly from that in healthy controls [14]. More recently, Drużdż et al. reported that patients with cervical dystonia had shorter CuSP duration and end latency than healthy controls, whereas onset latency did not differ significantly between the groups [15]. Taken together, these inconsistent findings suggest that silent-period characteristics in dystonia may vary across clinical populations and methodologies, and it remains unclear whether spinal inhibitory function assessed by the CuSP can be dynamically modulated by interventions targeting the broader motor network.

GPi-DBS is an established treatment for medically refractory dystonia and provides a valuable physiological model for probing disease mechanisms *in vivo* [16]. By modulating basal ganglia output and, in turn, downstream sensorimotor network dynamics, GPi-DBS creates an opportunity to examine whether spinal inhibitory function can change in association with therapeutic modulation of the broader motor network.

Accordingly, we examined CuSP parameters in patients with cervical dystonia before and after bilateral GPi-DBS and compared them with those of age- and sex-matched healthy controls. We sought to determine whether CuSP parameters differ between patients with cervical dystonia and healthy controls and whether these parameters change during the early postoperative period while GPi-DBS is active. We hypothesized that CuSP measures would show perioperative changes following GPi-DBS, consistent with the possibility that spinal inhibitory processing is dynamically influenced by changes within the broader sensorimotor network.

## Methods

We included 18 patients with cervical dystonia (mean age: 56.0 ± 11.8 years; male/female: 13/5) with two etiologies (16 idiopathic and 2 tardive) and a mean disease duration of 9.0 ± 8.0 years. Detailed clinical characteristics of these patients are shown in Tables 1a and 1b.

**Table 1a:** Clinical characteristics and timing of assessments in patients with cervical dystonia.


NO.	AGE (YEARS)	SEX	ETIOLOGY	DYSTONIA TYPE	DYSTONIA DURATION (YEARS)	Pre-DBS TWSTRS score (day)	Pre-DBS CuSP (day)	Post-DBS TWSTRS score (day)	Post-DBS CuSP (day)

1	25	M	Idiopathic	Phasic	16	47 (–4)	–5	25 (+11)	+9

2	39	M	Tardive	Tonic	6	25 (–5)	–7	24 (+9)	+10

3	46	M	Idiopathic	Tonic	1	47.75 (–3)	–4	38 (+10)	+4

4	48	M	Tardive	Phasic	10	–	–3	–	+9

5	50	M	Idiopathic	Combined	2	56.75 (–4)	–3	32.5 (+8)	+7

6	55	M	Idiopathic	Combined	5	35.5 (–4)	–5	32.75 (+9)	+13

7	56	M	Idiopathic	Tonic	20	36.75 (–8)	–8	22 (+13)	+20

8	57	M	Idiopathic	Combined	7	38 (–4)	–3	21 (+10)	+10

9	58	M	Idiopathic	Tonic	3	15 (–4)	–4	0 (+8)	+10

10	63	M	Idiopathic	Combined	4	28 (–3)	–3	29 (+11)	+9

11	64	M	Idiopathic	Tonic	11	42 (–3)	–4	41.75 (+15)	+15

12	71	M	Idiopathic	Combined	6	39 (–3)	–4	38.5 (+16)	+9

13	71	M	Idiopathic	Combined	31	–	–3	–	+40

14	50	F	Idiopathic	Tonic	2	47.75 (–3)	–4	13 (+17)	+16

15	56	F	Idiopathic	Combined	10	46.75 (–3)	–3	42 (+17)	+15

16	65	F	Idiopathic	Tonic	10	41.5 (–10)	–10	32 (+9)	+10

17	65	F	Idiopathic	Tonic	12	51 (–5)	–5	58.5 (+233)*	+22

18	69	F	Idiopathic	Combined	3	59.5 (–5)	–4	30.5 (+23)	+23


Values in parentheses indicate the timing of TWSTRS assessment relative to the date of DBS surgery (day 0). The timing of CuSP assessment is similarly expressed as days relative to surgery. Negative and positive values indicate assessments performed before and after surgery, respectively. TWSTRS assessments were not available for patients 4 and 13. *In patient 17, the postoperative TWSTRS assessment was performed 233 days after surgery, whereas the postoperative CuSP assessment was performed 22 days after surgery. Because of this substantial temporal discrepancy, patient 17 was excluded from the analysis of the relationship between changes in TWSTRS scores and CuSP measures. This analysis was therefore performed in the remaining 15 patients. Abbreviations: CuSP, cutaneous silent period; DBS, deep brain stimulation; TWSTRS, Toronto Western Spasmodic Torticollis Rating Scale.

**Table 1b:** Medications in patients with cervical dystonia.


NO.	MEDICATION (DOSE PER DAY)

1	–

2	Aripiprazole 12 mg, Lithium 600 mg, Loflazepate 2 mg, Escitalopram 10 mg, Trihexyphenidyl 6 mg, Clonazepam 3 mg, Bromazepam 6 mg

3	Trihexyphenidyl 10 mg

4	Lamotrigine 400 mg, Clonazepam 1.5 mg, Haloperidol 3.375 mg, Valproate 400 mg

5	Clonazepam 2 mg, Eperisone 100 mg, Alprazolam 0.4 mg, Etizolam 0.5 mg

6	–

7	–

8	Bromazepam 5 mg

9	Pregabalin 300 mg, Tramadol 75 mg, Lithium 300 mg, Bromazepam 12 mg, Alprazolam 1.2 mg, Flunitrazepam 2 mg

10	–

11	–

12	–

13	Clonazepam 0.5 mg, Arotinolol 10 mg

14	Clonazepam 1 mg

15	Sertraline 100 mg, Brexpiprazole 1 mg, Quetiapine 50 mg

16	–

17	Clonazepam 1.5 mg

18	Escitalopram 10 mg


Medications and doses represent the daily treatment regimen at the time of perioperative assessment. A dash indicates no medication.

Cervical dystonia was the predominant and consistently documented clinical manifestation in all patients. Extracervical dystonia, including facial or upper-limb involvement, was not systematically assessed or documented, particularly when symptoms were intermittent; therefore, extracervical involvement could not be reliably characterized in all patients.

All patients were admitted to our institution for bilateral GPi-DBS implantation (Table 2). Age- and sex-matched controls (mean age: 56.3 ± 12.3 years; male/female: 13/5; n = 18) were recruited for comparison with the patient group. All participants provided written informed consent before enrollment.

**Table 2:** GPi-DBS parameters in individual patients.


PATIENT	RIGHT GPi DBS			LEFT GPi DBS			STIMULATOR	ELECTRODE
	
	AMPLITUDE	PULSE WIDTH (μS)	FREQUENCY (Hz)	AMPLITUDE	PULSE WIDTH (μS)	FREQUENCY (Hz)		

1	2.5 V	60	130	2.5 V	60	130	Activa RC, Medtronic	3389

2	3.6 mA	60	130	3.5 mA	60	130	Vercise Genus RC, Boston	Twist

3	5.0 mA	60	130	4.8 mA	60	130	Vercise Genus RC, Boston	Directional

4	4.5 mA	60	130	4.5 mA	60	130	Vercise Genus RC, Boston	Directional

5	3.5 mA	60	130	3.5 mA	60	130	Vercise Gevia, Boston	Directional

6	2.5 mA	200	130	2.5 mA	200	130	Vercise Gevia, Boston	Directional

7	1.1 V	60	130	1.1 V	60	130	Activa RC, Medtronic	3389

8	7.0 mA	60	130	7.0 mA	60	130	Vercise Gevia, Boston	Directional

9	4.4 mA	60	130	5.9 mA	60	130	Vercise Genus RC, Boston	Directional

10	4.0 mA	60	130	4.0 mA	60	130	Vercise Genus RC, Boston	Twist

11	4.7 mA	60	130	4.7 mA	60	130	Vercise Genus RC, Boston	Directional

12	3.0 mA	60	130	3.0 mA	60	130	Vercise Genus RC, Boston	Directional

13	4.5 mA	60	130	4.5 mA	60	130	Vercise Genus RC, Boston	Directional

14	5.9 mA	60	130	5.9 mA	60	130	Vercise Genus RC, Boston	Directional

15	5.0 mA	60	130	5.0 mA	60	130	Vercise Genus RC, Boston	Twist

16	4.0 mA	60	130	4.0 mA	60	130	Vercise Genus RC, Boston	Directional

17	3.3 V	210	130	3.3 V	210	130	Activa RC, Medtronic	3389

18	2.7 mA	200	130	2.7 mA	200	130	Vercise Gevia, Boston	Directional


GPi, globus pallidus internus; DBS, deep brain stimulation.

This study was approved by the Institutional Ethics Committee of Fukuoka Mirai Hospital (approval date: June 1, 2022; approval no. 202206-1) and was conducted in accordance with the Declaration of Helsinki.

### Study Design

This single-center, exploratory observational study included repeated physiological measurements collected before and after DBS, as well as comparison with healthy controls. Patients underwent CuSP testing preoperatively, before implantation, and postoperatively during active GPi-DBS. GPi stimulation was initiated on the day after DBS implantation, and programming adjustments were subsequently performed according to the clinical response. Postoperative CuSP recordings were generally obtained during the postoperative hospitalization; in one patient (patient 13), the postoperative recording was obtained at an outpatient visit 40 days after surgery. GPi-DBS remained continuously ON throughout the postoperative CuSP recordings. The exact timings of the preoperative and postoperative CuSP assessments relative to DBS surgery for each patient are provided in Table 1a.

DBS programming was performed using a generally similar strategy across patients. Stimulation was initially programmed at a pulse width of 60 μs and a frequency of 130 Hz, and was subsequently optimized for each patient based on the clinical response. Stimulation amplitude and, when necessary, pulse widths were adjusted while monitoring the clinical effects. The stimulation parameters in use at the time of each postoperative CuSP recording for each patient are shown in Table 2.

The objectives were to determine whether CuSP responses differed between patients with cervical dystonia and healthy controls, and whether CuSP responses changed within patients after initiation of GPi-DBS.

CuSP recordings and signal analyses were performed by an investigator (EU) who was blinded to the Toronto Western Spasmodic Torticollis Rating Scale (TWSTRS) assessment results. TWSTRS assessments were performed independently by another physician (MY) and a nurse (EH), both of whom were unaware of the CuSP results.

### CuSP Recording Procedure

The CuSP method was similar to that in our previous reports [12,13]. The CuSP during isometric thumb contraction while delivering cutaneous electrical stimuli to the index finger was recorded. Ring electrodes were placed over the D2 interphalangeal joints to deliver constant square-wave electrical stimuli (0.5 ms), with the cathode and anode positioned proximally and distally, respectively. The sensory threshold for electrical stimulation was determined separately immediately before each CuSP recording session, and the stimulus intensity was set at 15 times the session-specific sensory threshold (15ST), consistent with our previously established CuSP protocol [12,13]. Therefore, although the absolute stimulus current could vary across participants and recording sessions, the relative stimulus intensity was consistently standardized at 15ST. This stimulation was tolerated by all participants.

Voluntary electromyographic activity was recorded using surface Ag/AgCl electrodes on the abductor pollicis brevis (APB), with the reference electrode placed at the thumb interphalangeal joint. Sensitivity was set at 200 μV/div, with a 10–5000-Hz bandpass filter. Electrical stimuli were delivered manually at interstimulus intervals of approximately 2–3 s. Before CuSP recording, patients were asked to perform a maximum voluntary isometric contraction of the APB muscle while viewing the electromyography (EMG) waveform on the monitor. They were then instructed to maintain voluntary thumb abduction at approximately 50% of the maximum EMG amplitude using the displayed EMG waveform as visual feedback. Full-wave rectification was performed, and electromyographic activity across 10 trials was averaged. The analysis window included 30–90-ms prestimulus and 360–420-ms poststimulus periods [12,13]. CuSP recordings were performed bilaterally. For each participant, the values obtained from the right and left sides were averaged, and the bilateral mean values were used as the individual onset latency, end latency, and duration for statistical analyses.

CuSP recordings were performed 4.6 ± 1.9 days preoperatively and 13.9 ± 8.3 days postoperatively (n = 18) (Table 1a).

### CuSP Signal Analysis

The CuSP latency analysis method was the same as that previously reported [12,13]. The baseline level was defined as 80% of the amplitude between the prestimulus electromyographic trough and peak in the rectified trace. CuSP onset and end latencies were determined manually from the averaged rectified EMG trace. CuSP onset was defined as the first drop in the electromyographic trace below baseline, and CuSP end was defined as the point at which the trace returned to baseline. CuSP duration was defined as the latency from CuSP onset to CuSP end.

Recordings with substantial artifacts or insufficient signal quality that prevented reliable identification of CuSP onset or end according to these predefined criteria were considered unsuitable for analysis. Patients with such recordings were excluded before establishment of the final study cohort.

The investigator performing the CuSP analysis was unaware of the TWSTRS assessment results but was not blinded to whether the recording was obtained preoperatively or postoperatively.

### Clinical Assessment

Clinical dystonia severity was assessed using the TWSTRS. All three TWSTRS subscales (Severity, Disability, and Pain) were assessed, and their sum was used as the TWSTRS total score for analysis. The exact timing of the preoperative and postoperative TWSTRS assessments relative to DBS surgery for each patient is provided in Table 1a.

### Statistical Analysis

Continuous variables are reported as means ± standard deviations. CuSP end latency was prespecified as the primary endpoint because it provides a direct measure of the termination of the silent period and is less susceptible to variability in onset determination [12,17]. CuSP onset latency and duration were analyzed as secondary endpoints. CuSP duration was not selected as the primary endpoint because it is mathematically derived from onset and end latencies; hence, it does not represent an independent physiological measure. Within-patient preoperative vs. postoperative comparisons were conducted using the two-sided Wilcoxon signed-rank test. Inter-group comparisons with healthy controls were conducted using two-sided Welch’s t-tests to avoid assuming equal variance. Preoperative and postoperative TWSTRS scores were compared using a paired t-test. Associations between changes in the CuSP parameters and changes in TWSTRS scores were examined using Pearson’s correlation coefficients. For these analyses, improvement in the TWSTRS score was defined as the preoperative minus postoperative score, whereas change in each CuSP parameter was defined as the postoperative minus preoperative value. Because a single primary endpoint was prespecified, no multiplicity adjustment was applied to the primary analysis. Secondary endpoint analyses were interpreted as supportive or exploratory. Two-sided P-values of less than 0.05 were considered statistically significant. All analyses were conducted using Statcel4 software (Ohmsha, Tokyo, Japan). No formal a priori sample size calculation was performed because this was an exploratory observational study based on consecutively available patients undergoing GPi-DBS for cervical dystonia at a single center.

### Use of Artificial Intelligence Tools

ChatGPT (OpenAI) was used for English-language editing, readability enhancement, and general assistance with manuscript preparation. It was not used to perform statistical calculations or generate study results. All statistical analyses, interpretation of the findings, and scientific conclusions were independently verified and determined by the authors. All final content was reviewed and approved by the authors.

## Results

### Participant Characteristics

Eighteen patients with cervical dystonia completed both preoperative and postoperative CuSP assessments. Eighteen age- and sex-matched healthy participants served as controls. Clinical characteristics, DBS programming parameters, and summary electrophysiological data are presented in Tables 1, 2, 3.

### Primary Endpoint: CuSP End Latency

As shown in Table 3, preoperative CuSP end latency did not differ significantly from control values (137.8 ± 16.3 ms vs. 144.8 ± 14.5 ms). At the postoperative assessment during active bilateral GPi-DBS, CuSP end latency was significantly increased compared with the preoperative value (145.1 ± 17.2 ms vs. 137.8 ± 16.3 ms; P = 0.0090). Postoperative CuSP end latency did not differ significantly from control values (145.1 ± 17.2 ms vs. 144.8 ± 14.5 ms).

**Table 3:** Mean values of CuSP measurements for patients (N = 18) and controls (N = 18).


VARIABLE	PATIENTS (N = 18) Pre-DBS	PATIENTS (N = 18) Post-DBS	CONTROLS (N = 18)	Pre-DBS vs. Post-DBS p†	Pre-DBS vs. Controls p‡	Post-DBS vs. Controls p‡

CuSP onset (ms)	63.7 (15.8)	61.4 (16.5)	60.2 (13.1)	0.184	0.464	0.806

CuSP end latency (ms)	137.8 (16.3)	145.1 (17.2)	144.8 (14.5)	0.0090	0.180	0.958

CuSP duration (ms)	74.0 (25.8)	83.7 (24.4)	84.6 (16.1)	0.0084	0.149	0.892


Values are presented as means (standard deviations). Abbreviations: CuSP, cutaneous silent period; DBS, deep brain stimulation. † Wilcoxon signed-rank test (two-sided). ‡ Welch’s t-test (two-sided).

### Secondary Endpoints

CuSP duration was significantly increased at the postoperative assessment during active DBS (83.7 ± 24.4 ms postoperatively vs. 74.0 ± 25.8 ms preoperatively; P = 0.0084) and was similar to control values (84.6 ± 16.1 ms) (Table 3).

CuSP onset latency demonstrated no significant within-patient or inter-group differences (preoperative: 63.7 ± 15.8 ms; postoperative: 61.4 ± 16.5 ms; controls: 60.2 ± 13.1 ms) (Table 3).

### Clinical Outcomes

TWSTRS assessments were unavailable in patients 4 and 13. Patient 17 had a postoperative TWSTRS assessment 233 days after surgery, which was considered temporally too remote from the postoperative CuSP recording at 22 days for inclusion in the analysis of the relationship between changes in TWSTRS scores and CuSP measures (Table 1a).

TWSTRS assessments temporally appropriate for comparison with the perioperative CuSP recordings were available in 15 of the 18 patients. TWSTRS scores improved significantly from 40.4 ± 11.7 preoperatively to 28.1 ± 11.3 postoperatively (paired t-test, P = 0.0009). In these 15 patients, TWSTRS assessments were performed 4.4 ± 2.0 days before and 12.4 ± 4.3 days after surgery, while CuSP recordings were obtained 4.7 ± 2.1 days before and 12.0 ± 5.0 days after surgery.

### Relationship Between Electrophysiological and Clinical Changes

No significant correlation was observed between the improvement in TWSTRS score (preoperative minus postoperative score) and the change in CuSP end latency (postoperative minus preoperative value) in the 15 patients with temporally corresponding TWSTRS and CuSP assessments (Pearson’s r = –0.486, P = 0.066). Similarly, changes in CuSP duration (r = –0.230; P = 0.410) and onset latency (r = –0.110; P = 0.697) were not significantly correlated with improvements in the TWSTRS score.

## Discussion

An interesting finding of the present study was that both CuSP end latency and duration were significantly increased at the early postoperative assessment during active GPi-DBS compared with preoperative values, whereas onset latency remained unchanged (Table 3, Figure 1). Although neither preoperative end latency nor duration differed significantly from those of healthy controls, both parameters were numerically shorter preoperatively and longer at the postoperative assessment. In particular, mean end latency increased from 137.8 ms preoperatively to 145.1 ms postoperatively, closely approximating the value of 144.8 ms observed in healthy controls. Similarly, CuSP duration increased from 74.0 to 83.7 ms, compared with 84.6 ms in controls. Thus, the principal finding of the present study is not the presence of a baseline abnormality relative to healthy controls, but rather a significant early postoperative within-patient change in selected CuSP parameters measured during active GPi-DBS.

**Figure 1:**
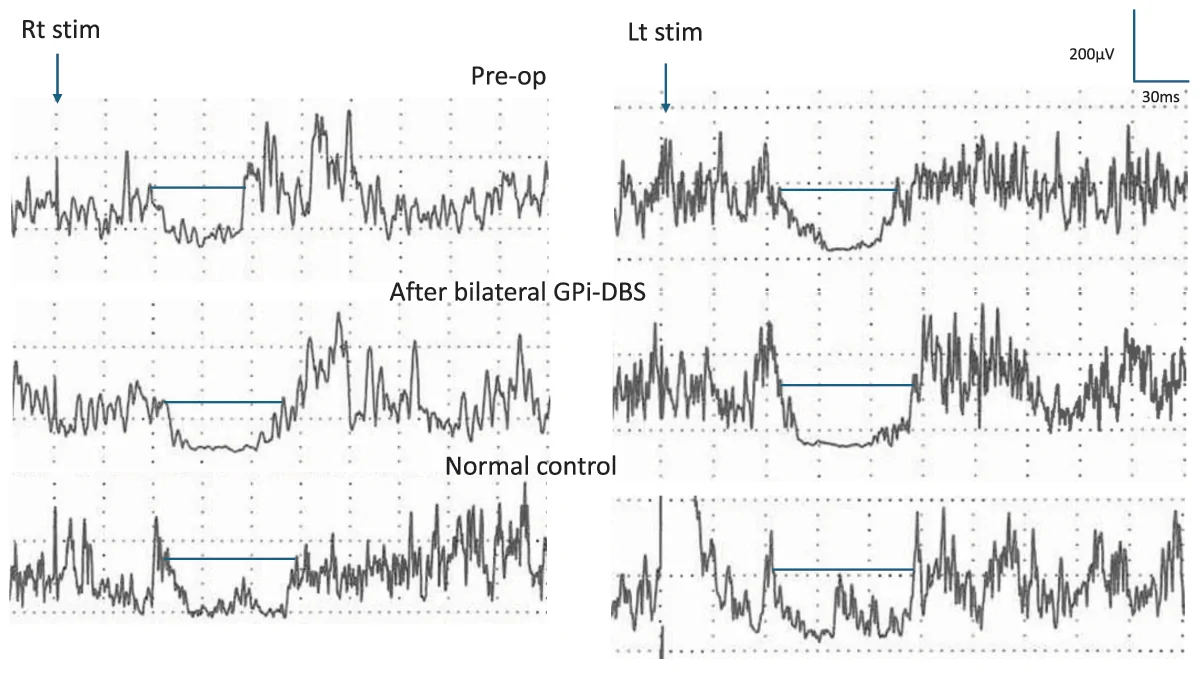
Representative electromyographic recordings illustrating the cutaneous silent period in a typical patient with cervical dystonia (patient #6) before surgery and at the early postoperative assessment during active GPi-DBS, and in a healthy control participant (a 58-year-old male).

This pattern is of particular interest in light of the recent findings of Drużdż et al., [15] who reported significantly shorter CuSP duration and end latency in patients with cervical dystonia than in healthy controls. Although our preoperative patient-control comparisons did not reach statistical significance, the numerical direction of the baseline differences was similar to that reported by Drużdż et al. The significant early postoperative within-patient prolongation observed in our study further raises the possibility that inhibitory sensorimotor mechanisms reflected in the later component of the CuSP may change during the early postoperative period while GPi-DBS is active.

Importantly, this interpretation should be regarded as hypothesis-generating rather than evidence of normalization because the preoperative differences from controls were not statistically significant, and neither preoperative nor postoperative values differed significantly from those of healthy controls. Nevertheless, the selective early postoperative changes in end latency and duration, with relative preservation of onset latency, suggest that mechanisms involved in the maintenance or termination of the CuSP may be more susceptible to change during the perioperative period than those involved in its initiation.

The early postoperative increase in CuSP end latency observed during active GPi-DBS is also noteworthy because it contrasts with a previous report that found no significant CuSP latency change in patients with cervical or generalized dystonia approximately 2 h after DBS was switched off [10]. This discrepancy may reflect differences in the timing and conditions of DBS assessment, as well as differences in dystonia phenotype, disease severity, disease duration, treatment history, background muscle activation, or methodological approaches to CuSP measurement [8,9,10]. In the present study, postoperative CuSP measurements were obtained during active GPi-DBS in the early postoperative period, whereas the previous study examined the acute effect of short-term DBS deactivation. Therefore, these different experimental paradigms may probe different aspects or time scales of physiological modulation. Taken together, the available findings suggest that CuSP responses in dystonia are not uniform and may depend on both clinical and experimental conditions.

The early postoperative within-patient changes observed during active GPi-DBS suggest that CuSP characteristics are not necessarily fixed but may vary in association with changes in the broader motor network during the perioperative period. GPi-DBS is known to modulate basal ganglia output and influence thalamocortical and broader motor network dynamics [1,2,3,16]. Thus, active stimulation represents one plausible contributor to the observed CuSP changes; however, the present study design does not allow its specific contribution to be separated from the effects of surgery, electrode implantation, or other perioperative factors. Therefore, these findings are consistent with, but do not establish, the possibility that spinal inhibitory processing is influenced by descending modulation from supraspinal motor networks.

This interpretation is compatible with the current view of dystonia as a network disorder characterized by altered inhibitory processing across multiple levels of the motor system [1,2,3,16]. Previous studies have reported abnormalities in intracortical inhibition, surround inhibition, and brainstem excitability [1,3,4,5,6,7]. Our findings raise the hypothesis that spinal inhibitory processing, as assessed by the CuSP, may be dynamically influenced by changes within the broader motor network; however, this possibility requires confirmation in studies specifically designed to distinguish stimulation-related effects from other perioperative influences.

The precise neural pathways linking supraspinal network activity to spinal inhibitory interneuronal circuits remain unclear. One plausible mechanism involves descending influences of pallidal activity on brainstem motor networks. The GPi is a major output nucleus of the basal ganglia and is anatomically and functionally connected with brainstem motor regions, including the pedunculopontine region. Pallidal and nigral projections to the pedunculopontine nucleus, which in turn projects to the pontomedullary reticular formation, have been demonstrated in experimental studies [18,19]. The pontomedullary reticular formation gives rise to reticulospinal pathways that can modulate spinal interneuronal circuits. Accordingly, a pallido-brainstem-reticulospinal network represents one plausible pathway through which pallidal stimulation could be associated with changes in spinal inhibitory processing. Consistent with this possibility, Tisch et al. reported a progressive increase in forearm reciprocal inhibition after pallidal DBS in patients with dystonia [20], suggesting time-dependent reorganization of spinal inhibitory control. In contrast, another study found no acute DBS ON/OFF effect on CuSP, despite changes in cortical inhibition [10], suggesting that spinal effects may depend on the time course of stimulation and network plasticity rather than reflecting an immediate direct effect of DBS.

The present CuSP findings should not be interpreted as direct evidence of increased gamma-aminobutyric acid (GABA)ergic inhibition within the spinal cord. The CuSP is mediated predominantly by spinal inhibitory interneuronal circuits; however, its duration cannot be attributed to simple GABAergic or glycinergic transmission alone, and supraspinal modulation may also contribute [21]. High-frequency DBS can exert different effects on local somatic activity and axonal output, thereby modulating activity across connected neural networks [22]. Therefore, the observed CuSP changes may reflect network-level modulation of descending control of spinal inhibitory circuits rather than a direct local GABAergic effect. Stimulation parameters may also be relevant because amplitude, pulse width, frequency, and electrode contact selection influence the spatial extent and neural elements activated by DBS [22]. However, the present study was not designed to assess parameter-specific effects on spinal inhibitory circuits, and the relationship between GPi-DBS programming and CuSP changes requires further investigation.

The lack of significant correlations between changes in CuSP parameters and improvement in TWSTRS scores warrants consideration. Clinical rating scales quantify overt motor symptoms, whereas CuSP may reflect physiological processes that are not directly captured by symptom severity scores. However, the absence of a significant association in the present study means that our findings do not establish CuSP as a marker of clinical severity or treatment response. The relatively small number of patients included in the correlation analyses may also have limited the ability to detect such relationships. Longitudinal studies with repeated physiological and clinical assessments will be necessary to determine whether changes in CuSP track changes in dystonia severity over time.

The relatively early postoperative improvement in TWSTRS scores should be interpreted cautiously because the clinical response to GPi-DBS may evolve over different time courses according to dystonia phenomenology, and stimulation parameters were still being clinically adjusted at the time of assessment. Although tonic, phasic, and combined phenotypes were documented, the sample size was insufficient for a meaningful phenotype-specific analysis of the early clinical response.

## Limitations

This study had some limitations. First, the sample size was modest, reflecting the limited number of patients undergoing GPi-DBS for cervical dystonia at a single center. This modest sample size may have limited the statistical power of the secondary and correlation analyses. Second, CuSP onset and end latencies were determined manually by an investigator who was not blinded to the preoperative or postoperative status of the recordings, and formal intra- or inter-rater reliability testing was not performed; therefore, potential measurement bias and variability cannot be excluded. Third, extracervical dystonias were not systematically assessed in all patients. Therefore, we could not reliably classify all patients as having isolated cervical dystonia or determine whether intermittent facial, upper-limb, or other extracervical dystonias were present. Fourth, the inclusion of two patients with tardive dystonia may have introduced etiological heterogeneity. Fifth, several patients were receiving concomitant medications that could potentially influence spinal excitability and CuSP parameters. For example, tramadol and escitalopram have been reported to prolong the CuSP [23,24]. The regular medication regimen of each patient remained unchanged between the preoperative and postoperative CuSP assessments, and no additional sedative or analgesic medication beyond the patients’ regular medication regimen was administered on the day of the postoperative CuSP assessment. Nevertheless, we could not statistically control for the effects of individual medications because of the small sample size and heterogeneous treatment regimens. Therefore, medication-related effects on the absolute CuSP values or on the magnitude of the observed postoperative changes cannot be completely excluded, and the present findings should be interpreted with caution. Finally, postoperative CuSP assessments were performed during the early postoperative period while stimulation parameters were being clinically adjusted, without a corresponding DBS-OFF assessment or a delayed assessment after stabilization of DBS programming. Therefore, the observed preoperative-to-postoperative changes cannot be attributed specifically to GPi stimulation and may also reflect effects related to surgery and electrode implantation, including a possible microlesion effect, as well as other perioperative factors. Although both the preoperative and postoperative CuSP assessments were performed during hospitalization, potential differences in perioperative conditions cannot be completely excluded. Furthermore, because DBS-OFF measurements were not performed, the reversibility of the observed physiological changes and their direct dependence on active stimulation could not be established. Accordingly, the present findings should be interpreted as early postoperative changes in CuSP measured during active GPi-DBS rather than as evidence of a stimulation-specific or reversible DBS effect. Future prospective studies incorporating DBS ON/OFF comparisons after stabilization of stimulation parameters, as well as longer-term follow-up, are needed to determine the specific contribution of stimulation and whether the observed CuSP changes persist or evolve over time.

## Conclusions

CuSP end latency and duration were significantly increased at the early postoperative assessment during active GPi-DBS in patients with cervical dystonia, whereas onset latency remained unchanged. Neither preoperative nor postoperative CuSP measures differed significantly from those of healthy controls. These findings indicate that selected CuSP parameters can change dynamically during the early postoperative period, even in the absence of a demonstrable baseline abnormality relative to healthy controls. Although active GPi stimulation may have contributed to these changes, the present study cannot distinguish stimulation-specific effects from those of surgery, electrode implantation, or other perioperative factors. Further longitudinal studies incorporating DBS ON/OFF comparisons after stabilization of stimulation parameters are needed to clarify the specific contribution of GPi stimulation to the temporal dynamics of CuSP.
